# Professionals’ perspectives on barriers to improving psychosocial care for adolescents with emotional and behavioural problems – a concept mapping case study

**DOI:** 10.3389/ijph.2026.1610058

**Published:** 2026-10-07

**Authors:** Katerina Paclikova, Lucia Bosakova, Zuzana Dankulincova Veselska, Peter Tavel, Sijmen A. Reijneveld

**Affiliations:** 1 Olomouc University Social Health Institute, Palacky University in Olomouc, Olomouc, Czechia; 2 Department of Community and Occupational Medicine, University Medical Center Groningen, University of Groningen, Groningen, Netherlands; 3 Department of Health Psychology and Research Methodology, Faculty of Medicine, PJ Safarik University in Kosice, Kosice, Slovakia

**Keywords:** adolescents, concept mapping, emotional and behavioral problems, mental health services, psychosocial care, barriers, mental health gap

## Abstract

**Objectives:**

Rates of emotional and behavioural problems (EBP) in adolescence are high and rising, increasing pressure on psychosocial care systems. Professionals working in these systems have excellent insights in the measures needed. Of these, the most urgent and implementable ones have already been elucidated. Our current aim was to identify measures and interventions to improve psychosocial care for adolescents with EBP that professionals perceived as highly urgent but not yet feasible to implement, revealing potential barriers for implementation.

**Methods:**

We used Concept mapping, a participatory mixed-method approach, combining qualitative data collection with quantitative analysis. Overall, 33 professionals from 17 institutions participated.

**Results:**

Participants proposed 43 measures/interventions, that could be categorized in five clusters. Of these, 13 were identified as highly urgent but not feasible. Most belonged to clusters related to school system changes, improvement of transparency and functioning of the system of care, and legislative conditions for adolescents with EBP.

**Conclusion:**

Findings highlight implementation challenges related to school systems, workforce capacity, and system fragmentation. Addressing these challenges will require coordinated system-level approaches and long-term commitment to improve adolescent psychosocial care.

## Introduction

The worsening mental health of adolescents is an important public health challenge [1]. Emotional and behavioural problems (EBP) are among the most common health issues in childhood and adolescence with estimates suggesting that in Europe 5.7%–36.7% of adolescents are affected [2]. The prevalence of EBP among adolescents in Slovakia mirrors global trends. The nationally representative HBSC study similarly reports a prevalence rate of 10%–20% among adolescents aged 11–15, with older girls being at a higher risk [3]. Adolescent mental health problems often persist throughout adolescence into adult life, have both short and long-term impacts on health and represent a major burden of disease [4–6]. Therefore, to reduce the burden of mental health problems, effective delivery of care is of the great importance.

Care for adolescents with EBP typically regards a system, i.e., an interconnected network of individuals and institutions involved in supporting adolescents with EBP and their families. It encompasses professional practices, established relationships, collaborative connections, and opportunities for mutual cooperation. This comprehensive system spans the full continuum of services, from parental and counselling support to psychological, social, and psychiatric care [7]. Previous research already confirmed a wide range of barriers that hinder the effective provision of psychosocial care [8, 9] and efforts are ongoing to improve the performance of the care system and its ability to respond to client needs [10].

To effectively improve this system of psychosocial care, we need to identify not only the potentially effective measures and interventions, but also the potential barriers in their implementation. Involvement of key stakeholders, i.e., participatory approach, is pivotal to identify these implementation barriers, as it helps to combine the theoretical experience of researchers with the practical experience of people from practice into a mutually reinforcing partnership. This helps to overcome the gap between theory and practice [11]. Key sources of information in this process regard care professionals, who interact with clients daily and possess invaluable insights into practical challenges and systemic shortcomings. These professionals with their perspective deeply rooted in their everyday practice can evaluate both the urgency as well as feasibility of proposed measures to establish priorities effectively and reveal potentially problematic areas that need to be carefully addressed.

Bosakova et al. [10] previously reported on measures perceived as both highly urgent and highly feasible, representing priorities for improving the system of care. The present case study using concept mapping approach builds on this work by focusing specifically on measures perceived as highly urgent but not yet feasible to implement. While these measures are less directly actionable, they reflect areas where the need for change is strongly recognised but difficult to achieve in practice. Focusing on this group of measures can lead to a better understanding of barriers that may limit the implementation of improvements in psychosocial care. We therefore aimed to identify measures and interventions focused on improvement of the psychosocial care for adolescents with EBP which implementation were considered by professionals as urgent but not yet feasible, revealing potential barriers for implementation.

## Methods

### Design

We employed Concept Mapping (CM), an integrated mixed-method approach combining qualitative data collection with quantitative data analysis. This approach enables a diverse group of stakeholders to qualitatively articulate their ideas and subsequently quantitatively sort and rate them, allowing for statistical analysis and visualisation of relationships between the identified measures. CM is a robust method for assessing how study participants cluster their conceptual understanding of a given topic by constructing a conceptual framework and visually displaying the clustering [12]. It facilitates the mapping of complex concepts that may not be explicitly identified by participants [13]. Moreover, CM supports a participatory approach by actively involving stakeholders and empowering specific groups, such as frontline workers, while presenting results in a manner accessible to diverse audiences.

### Sample

The CM case study was conducted in the eastern part of Slovakia, primarily in Kosice, the country’s second-largest city. This location was selected because of the availability of comprehensive care services for adolescents with EBP and the research team’s established personal contacts in the area, which significantly facilitated field access. To ensure a diverse range of perspectives and enhance the generalizability of the conceptual framework, we recruited participants according to the guidelines of [12, 14]. A purposive sampling technique was utilized to select stakeholders representing various hierarchical levels across the three main care categories: preventive-counselling, social care, and healthcare.

A total of 40 stakeholders were initially approached, of whom 33 consented to participate (response rate: 82.5%). Non-participants were predominantly from managerial positions, citing workload as the primary reason for declining participation. The final sample included representatives from 17 institutions, comprising psychologists, special needs educators, educational counsellors, social workers, and child psychiatrists. All participants were female, reflecting the predominantly feminized nature of the sectors involved. Participants' ages ranged from 25 to 65 years. The sample sizes at each stage of CM (brainstorming: 25, sorting/rating: 33, interpretation: 23) were deemed sufficient to meet statistical validity and reliability criteria [15]. Table 1 provides an overview of the number and type of participants (and institutions) based on their participation in particular phases of the CM case study (brainstorming, sorting/rating, interpretation), see next section.

**TABLE 1:** Number and type of participants of the CM case study (Slovakia, 2018–2019).

​	Counselling care			Social care			Healthcare		
CM steps	B	S/R	I	B	S/R	I	B	S/R	I
Number of institutions	7	7	7	6	7	6	4	5	5
Number and type of providers	10	11	9	10	16	8	5	6	6
Psychologist	5	6	4	4	5	3	2	2	2
Child psychiatrist	-	-	-	-	-	-	2	3	3
Social worker	-	-	-	6	11	5	1	1	1
Special needs pedagogue	2	2	2	-	-	-	-	-	-
Teacher	2	2	2	-	-	-	-	-	-
Educational consultant	1	1	1	-	-	-	-	-	-

B–brainstorming, S/R–sorting/rating, I–interpretation.

### Procedure and analysis

The CM process was conducted between November 2018 and November 2019 and followed five structured steps as outlined by [14]: preparation, brainstorming, sorting and rating, analysis, and interpretation.

#### Preparation

We formulated the focus prompt (“What do you think needs to be done to improve the system of care for adolescents with emotional and behavioural problems in their favour?”) which aimed to elicit stakeholder opinions on improving the care system for adolescents with EBP, then conducted a pilot CM session with the research team (including researchers not directly involved in this study) to assess clarity and prepare for a facilitation of the subsequent brainstorming session. The pilot mapping session was conducted with members of the research team, several of whom had previous professional experience within the relevant care system, including clinical psychology and social work. This enabled the clarity and practical relevance of the terminology, instructions and mapping procedure to be considered from both research and care-provider perspectives.

#### Brainstorming

We organised a one-day workshop for brainstorming, in which participants were divided into four subgroups: preventive-counselling, social care, healthcare, and management. This subgroup division minimized power dynamics by separating frontline workers from managers. As EBP represents a broad concept, its meaning was clarified with participants before the focal question was presented. We used an empirical-descriptive understanding distinguishing between internalising problems (e.g., anxiety, sadness, withdrawal and psychosomatic complaints) and externalising problems (e.g., impulsivity, aggression, disruptiveness and antisocial behaviour), based on established literature [16–18]. Each subgroup generated statements related to the focus prompt (“What do you think needs to be done to improve the system of care for adolescents with emotional and behavioural problems in their favour?”), facilitated by trained moderators and supported by research assistants who documented the ideas. To facilitate active participation of all respondents, brainstorming was initially conducted in four smaller groups, which together generated 80 statements. Subsequently, in a group discussion with all participants we identified and merged statements that were identical, overlapping, or semantically similar. This process resulted in a final set of 43 distinct statements. This set was reviewed and approved by all participants present.

#### Sorting and rating

Participants sorted the 43 statements into piles based on conceptual similarity and labelled each pile. To ensure consistency, participants were instructed that (a) a statement could only belong to one pile, (b) no pile could contain just one statement, and (c) all statements could not be grouped together. They also rated each statement’s urgency and feasibility on a Likert scale from 1 (low) to 4 (high).

#### Analysis

Data quality was rigorously assessed, and only complete responses which aligned with the instructions were included in the analysis. We used GroupWisdom™ software to conduct multidimensional scaling and hierarchical cluster analysis, generating point and cluster maps. The optimal cluster solution (five clusters) was selected through iterative team discussions, based on both statistical considerations (fit indices) and participant preferences. Priority areas were identified by examining statements rated both highly urgent and feasible (Go-Zone). Barrier areas were identified by examining statements rated as highly urgent but not feasible (Gap-Zone). In line with the Group Concept Mapping approach described by Kane and Rosas [14], classification into the Gap-Zone was based on the overall mean ratings rather than predetermined absolute cut-off values on the 1–4 scales. Statements with urgency ratings above the overall mean and feasibility ratings below the overall mean were classified within the Gap-Zone, representing measures perceived as relatively more urgent but less feasible compared with the complete set of proposed measures.

#### Interpretation

The final interpretation workshop involved 23 participants who had been involved in previous steps. During this session, the research team and stakeholders collaboratively finalized the cluster labels and reviewed priority statements from the Go-Zone and Gap-zone maps, ensuring that the results accurately reflected the collective perspectives. During the final interpretation workshop, researchers and participants also identified broader areas and overarching themes connecting measures across the five clusters. These did not represent an additional statistical categorisation, but were used to organise the subsequent interpretation and discussion of the findings in a more integrated and less repetitive manner. This comprehensive and systematic approach ensured methodological rigor, transparency, and stakeholder engagement throughout the research process. In line with previous concept mapping studies, the present analysis focuses specifically on Gap-zone, defined as measures perceived as highly urgent but not feasible. Measures belonging to Go-zone (high urgency and high feasibility) have been analysed and reported in a previous study within the same project [10].

## Results

### Clusters of measures related to the improving the system of care for adolescents with EBP

Based on performed sorting, we obtained a final 5-cluster solution (see Figure 1): Cluster 1 - Increasing the competencies, possibilities and opportunities for providers and institutions in the system of care, Cluster 2 - Changes at the level of the school and the school system, Cluster 3 - Support for existing services targeting adolescents and families, Cluster 4 - Increasing the transparency and functionality of the system of care at the level of institutions and public administration, and Cluster 5 - Modification and creation of legislative conditions in the system of care for adolescents with EBP. The stress index of 0.233 indicates a strong fit between this cluster map and the data. Typically, the stress index in concept mapping should be between 0.10 and 0.35 according to [14].

**FIGURE 1:**
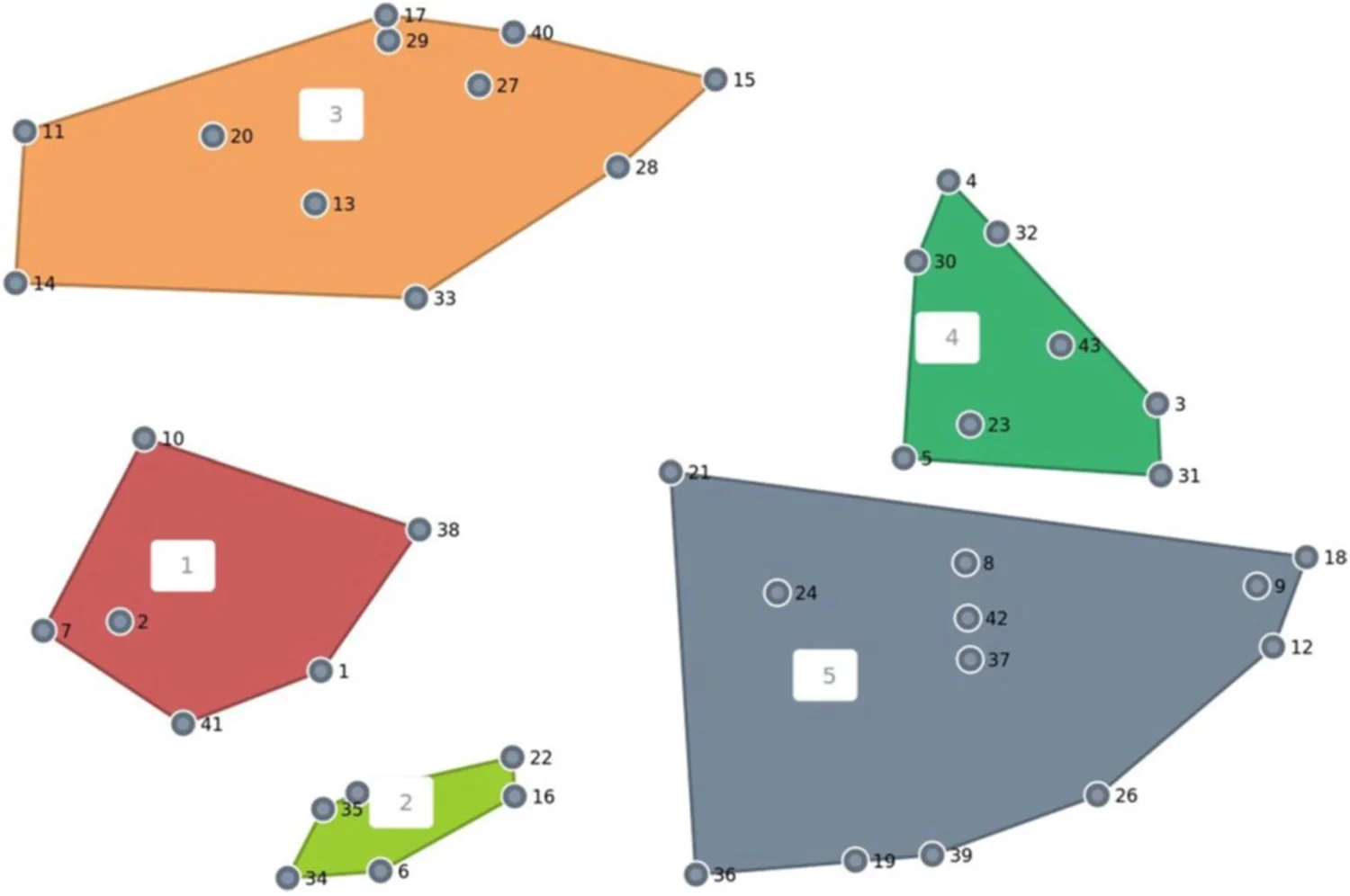
Clusters of measures: point map – final 5-cluster solution (Slovakia, 2018–2019). Note: Each dot represents a single measure proposed by participants. The closer the dots are to each other, the more participants sorted the measures into the same piles, i.e., regarded them as being part of the same concept. The size of the surface of a cluster indicates the degree to which its various contributing measures are related to each other. Created by Bosakova et al. (2024).

### Rating of individual measures by urgency and feasibility

Based on participants’ rating, out of 43 proposed measures and interventions, we identified 13 which were rated as highly urgent but not yet feasible and could, according to participants, expose serious barriers that may prevent action (Table 2). Identified measures belong to all 5 clusters. The highest number belongs to Cluster 2 (Changes at the level of the school and the school system), Cluster 4 (Increasing the transparency and functionality of the system of care at the level of institutions and public administration) and Cluster 5 (Modification and creation of legislative conditions in the system of care for adolescents with EBP). During the interpretation step all measures rated as urgent but not feasible were discussed by participants. General consensus was, that even though identified 13 measures spanned all 5 clusters all of them were considered as similarly problematic and thus less feasible due to the need of legislative and/or systematic changes done on the national level which require political will, additional financial and personal resources and takes time.

**TABLE 2:** Individual measures rated as the most urgent and least feasible divided by clusters (Slovakia, 2018–2019).

Measures/interventions by clusters	Urgency Mean	Feasibility Mean
*Cluster 1. Increasing the competencies, possibilities and opportunities for providers and institutions in the system of care*		
38. Creation of a functional methodological centre for the diagnostics of counselling work (psychologists, special educators) and establish a unified diagnostic structure	2.85	2.21
41. Improvement of the quality of practical training by involving care-providing institutions (dual education)	2.88	2.42
*Cluster 2. Changes at the level of the school and the school system*		
25. Creation of a motivated and financially rewarded teaching team at schools with a reduced teaching workload (including educational counsellors, prevention coordinators, and educational mediators)	2.88	2.30
34. Change in the way teachers are trained at the pre-graduate level	2.94	2.33
35. Reduction of the number of students in a class, taking into account the number of integrated students	3.27	2.45
*Cluster 3. Support for existing services targeting adolescents and families*		
40. Development of a proactive participatory tool to involve parents in addressing their child’s problems	3.00	2.45
33. Creation of a support system and services for affected groups - teachers, families, communities (including teacher and parent coaching)	2.88	2.39
*Cluster 4. Increasing the transparency and functionality of the system of care at the level of institutions and public administration*		
30. Creation of a comprehensive services under one roof with multidisciplinary teams	3.09	1.94
31. Ensuring continuity of state-funded projects	3.00	2.12
32. Establishing frameworks for parallel service provision within networks (networking)	2.85	2.25
*Cluster 5. Modification and creation of legislative conditions in the system of care for adolescents with EBP*		
8. Increased staffing capacity within the psychosocial care workforce and shorten waiting times	3.64	2.03
19. Reform of the entire educational system	3.42	1.45
42. Recognition of psychosocial care professionals as high-risk workforce and provide appropriate benefits	2.85	2.15

## Discussion

This case study aimed to identify measures and interventions focused on improvement of the psychosocial care for adolescents with EBP which implementation were considered by professionals as urgent but not yet feasible. From 43 measures proposed for the improvement of the system of care, 13 were rated as urgent but not feasible spanning all five clusters. During the final interpretation workshop with the respondents, these measures were discussed across clusters and later organised into three broader areas to facilitate an integrated interpretation of the findings in the Discussion: 1) school system change and support, 2) workforce capacity and resources, and 3) system fragmentation and coordination. In addition, problematic implementation of policy-based changes was identified as an overarching theme connecting these areas. These areas are closely interconnected and reflect challenges at multiple levels of the system, which is consistent with previous research on barriers in psychosocial care and access to services [7–9].

### School system change and support

A large proportion of the identified measures relates to the school system, including reform of educational structures, better teacher training, reduction of class sizes, and strengthening school-based teams. These findings confirm the central role of schools in addressing adolescents’ mental health. International evidence consistently identifies schools as key platforms for mental health promotion, prevention and early intervention [19]. However, the low ratings of feasibility that we found suggest that professionals perceive meaningful changes in schools as difficult to achieve. Findings from our previous qualitative study provide further insight into these challenges, showing that collaboration between schools and professionals is often limited, inconsistent, and dependent on individual initiative [8]. In addition, schools themselves often face capacity barriers, including high workload, lack of specialised staff and limited training in mental health [20–22]. Importantly, the identified measures should not be viewed solely as technical or organisational changes. The capacity of schools to support adolescent mental health is embedded in broader social and working conditions within the school environment [3, 21, 22]. Teacher workload and insufficient professional support may limit teachers’ capacity to recognise and respond to students’ difficulties [8, 20], while adverse school experiences, including schoolwork pressure and bullying, as well as socioeconomic inequalities, are associated with poorer mental health among adolescents [23, 24]. Thus, improving school-based mental health support requires attention not only to service structures and professional competencies, but also to the broader conditions in which students and school staff function. These findings suggest that meaningful school-based change requires broader systemic support and intersectoral cooperation. Beyond strengthening the capacity of schools to respond to adolescents experiencing EBP, emphasis could also be placed on universal mental health promotion as a complementary strategy. WHO [21] recommends universally delivered interventions covering social and emotional learning, with schools representing a suitable setting for such approaches. Universal school-based mental health promotion interventions can reach adolescents across populations while avoiding potential stigma. Evidence from Wang et al. [25] suggests positive effects of universal school-based interventions across various mental health domains. Such population-level approaches should not be viewed as substitutes for accessible specialised care, but as a part of a broader continuum of mental health promotion, prevention and treatment that may reduce the overall burden on already overstretched services.

### Workforce capacity and resources

Second, workforce-related measures were identified as highly urgent but not feasible. These include increasing staffing capacity, improving training, and recognising psychosocial care professionals as high-risk workforce. Such findings point to insufficient human resources as a main barrier in the system of care. Workforce shortages in mental healthcare are widely documented and represent a major barrier to service provision [8, 26]. An increasing demand for services, combined with limited capacity, places significant pressure on professionals and reduces the ability of systems to respond effectively. Workforce barriers are also closely linked to other barriers, such as limited time for collaboration, high workload, and reduced capacity to build relationships with clients, which may negatively affect the quality of care, continuity and interprofessional cooperation, as also reported in previous studies [8, 9, 27]. This suggests that strengthening workforce capacity is a necessary precondition for improving accessibility, continuity and quality of psychosocial care.

### System fragmentation

Third, a series of measures connected to system fragmentation were rated as highly urgent but not feasible, these focusing on improving coordination and continuity of care, and creation of multidisciplinary services. This suggests that fragmentation of services is perceived as one of the most critical, yet difficult to address, challenges. Our findings regarding insufficient interprofessional collaboration, limited information exchange, and unclear roles and responsibilities confirm previous research on relational barriers within psychosocial care systems [8, 28, 29]. The present case study extends these findings by identifying these barriers as areas perceived not only as highly urgent, but also as difficult to address in practice. At the system level, fragmentation has been identified as a major weakness of mental healthcare systems, particularly in contexts with limited integration across sectors [30]. Integrated and coordinated care models are therefore increasingly emphasised as a key strategy for improving accessibility, effectiveness and continuity of care [31, 32]. Importantly, fragmentation also affects relationships with clients. Previous findings indicate that discontinuity of care, involvement of multiple professionals and inconsistent communication may limit trust and engagement of adolescents and their families [8, 33]. These findings highlight that improving coordination and integration of services may be essential for building more continuous and trust-based care for adolescents and their families. To achieve this goal and to make intersectoral collaboration more feasible it is important to identify potential facilitators. According to systematic review by Cooper et al. [28], facilitators include regular communication across professional and services, joint interprofessional trainings, mutual understanding of services and professional roles, explicit protocols for intersectoral collaboration, designated link person and support from senior management. Together, these facilitators may help to move intersectoral collaboration from informal, person-dependent relationships towards more structured and sustainable forms of cooperation.

### Overarching: the problematic implementation of policy-based changes

The measures identified as urgently needed but not yet feasible covered three different areas, but they all have one thing in common: the problematic implementation of policy-based changes, which require political will, additional financial and/or personal resources and time. Similar implementation barriers have also been described in previous research focusing on psychosocial care systems and perspectives of professionals [8, 34, 35]. The present findings extend this knowledge by showing that such barriers are perceived by professionals as highly urgent, while at the same time difficult to address in practice. First, policy changes regarding the national system of psychosocial care for adolescents usually comprise several ministries. The collaboration between these ministries has been found to be problematic and time-consuming, and to require lobbying [7, 34]. Second, implementation was perceived as less feasible when proposed changes required substantial additional financial resources, personal capacities, or organizational support that were not sufficiently available or allocated [7, 35]. Finally, changes often do not align with the reality as perceived and needs as articulated by people in practice resulting in low feasibility. Thus, care providers may perceive little or no control over the legislative conditions as these can only be changed through political processes. This gap between policy-level changes and the realities of practice may be reduced through a greater recognition of experiential knowledge and more meaningful stakeholder participation. This could be done via bottom-up approaches that actively involve stakeholders directly affected by the system, including professionals, service users and communities, whose experiential knowledge can help identify barriers, needs and opportunities for improvement and enhance the relevance, acceptability and sustainability of interventions [11, 36]. Co-production with young people and their families may recognise them as partners rather than solely as recipients of care. However, it should be realised that meaningful participation requires supportive organisational structures, recognition of experiential knowledge as legitimate expertise, and opportunities for involvement beyond consultation, including policy development, implementation and evaluation [37]. Taken together, these findings suggest that implementation barriers are deeply embedded at the policy and system level and cannot be addressed without long-term intersectoral and political commitment.

### Strengths and limitations

One of the crucial strengths of this study was the participatory approach where professionals from the system of care were given the opportunity to provide their insights and propose their solutions along with potential pitfalls in the improvement of the system of care for adolescents with EBP. Another strength is the use of concept mapping, which combines qualitative and quantitative procedures and allows for a structured identification and prioritisation of measures perceived as urgent but difficult to implement.

Several limitations should also be considered. First, the concept mapping methodology is based on subjective assessments of participants and may be prone to social desirability, although we have reduced this risk by creating the subgroup of managers during the brainstorming to minimize the impact of power relations. Second, we only included women in this study, which may have influenced the identified priorities and perceptions of feasibility. Third, the study intentionally focused on care providers; consequently, the findings reflect provider perspectives and do not capture the experiences and priorities of adolescents with EBP and their families. Fourth, as the study was conducted in eastern Slovakia, the findings reflect this specific regional context and should not be considered representative of the national system of psychosocial care. Although EBP was explicitly defined and discussed with participants before brainstorming, their understanding of the concept was not formally assessed; therefore, some variation in interpretation cannot be excluded. Finally, data were collected in 2018–2019, prior to the COVID-19 pandemic, which substantially affected adolescent mental health and psychosocial care systems. As a result, professionals’ perceptions of the urgency and feasibility of the identified measures, as well as the broader policy context, may have changed since data collection.

### Implications

We found measures related to school systems, workforce capacity, and fragmentation of services to be perceived as highly urgent but not feasible, implying that improvement of psychosocial care for adolescents with EBP requires intersectoral collaboration across the education, social and health sectors, with clearly defined responsibilities. Policies should include explicit allocation of finances and personnel, otherwise implementation is likely to fail even if proposed measures or interventions are perceived as urgent. Incorporating practitioners’ perspectives and experiences can improve feasibility and relevance of the proposed changes, reducing the gap between policy and practice. Future research should examine these priorities from the perspectives of adolescents with EBP and their families, and across different regions of Slovakia.

### Conclusions

Our case study identified a gap between the urgency and feasibility of key measures to improve psychosocial care for adolescents with EBP. Our findings point to interconnected challenges related to school systems, workforce capacity, and system fragmentation, all of which require coordinated, resource-supported policy action. Addressing these challenges is essential for developing a more accessible, integrated, and responsive system of care will require coordinated, system-level approaches and long-term commitment.

## Data Availability

The data presented in this study are available on request from the corresponding author. The data are not publicly available due to privacy and ethical issues.
